# Dental pulp stem cells overexpressing hepatocyte growth factor facilitate the repair of DSS-induced ulcerative colitis

**DOI:** 10.1186/s13287-020-02098-4

**Published:** 2021-01-07

**Authors:** Ning Li, Yichi Zhang, Narayan Nepal, Guoqing Li, Ningning Yang, Haoyuan Chen, Qiuchi Lin, Xuechun Ji, Sijia Zhang, Shizhu Jin

**Affiliations:** Department of Gastroenterology and Hepatology, The Second Affiliated Hospital, Harbin Medical University, Harbin, Heilongjiang Province China

**Keywords:** Dental pulp stem cells, Hepatocyte growth factor, Ulcerative colitis

## Abstract

**Background:**

Ulcerative colitis (UC) is a chronic and recurrent disease without satisfactory treatment strategies. Dental pulp stem cell (DPSC) transplantation has been proposed as a potential therapy for UC. This study aimed to investigate the therapeutic effects of the rat hepatocyte growth factor (HGF) gene transduced into DPSCs for UC.

**Methods:**

The therapeutic effects of HGF-DPSCs transplanted intravenously into a rat model of UC induced by 5% dextran sulphate sodium (DSS) were compared with the other treatment groups (LV-HGF group, DPSCs group and GFP-DPSCs group). Immunofluorescence and immunohistochemistry were used to observe the localization and proliferation of HGF-DPSCs at the site of colon injury. The expression levels of inflammatory factors were detected by real-time quantitative PCR (RT-PCR) and western blotting. The oxidative stress markers were detected by ELISA. DAI scores and body weight changes were used to macroscopically evaluate the treatment of rats in each group.

**Results:**

Immunofluorescence and immunohistochemistry assays showed that HGF-DPSCs homed to colon injury sites and colocalized with intestinal stem cell (ISC) markers (Bmi1, Musashi1 and Sox9) and significantly promoted protein expression (Bmi1, Musashi1, Sox9 and PCNA). Anti-inflammatory cytokine (TGF-β and IL-10) expression was the highest in the HGF-DPSCs group compared with the other treatment groups, while the expression of pro-inflammatory cytokines (TNF-α and INF-γ) was the lowest. Additionally, the oxidative stress response results showed that malondialdehyde (MDA) and myeloperoxidase (MPO) expression decreased while superoxide dismutase (SOD) expression increased, especially in the HGF-DPSCs group. The DAI scores showed a downward trend with time in the five treatment groups, whereas body weight increased, and the changes were most prominent in the HGF-DPSCs group.

**Conclusions:**

The study indicated that HGF-DPSCs can alleviate injuries to the intestinal mucosa by transdifferentiating into ISC-like cells, promoting ISC-like cell proliferation, suppressing inflammatory responses and reducing oxidative stress damage, which provides new ideas for the clinical treatment of UC.

## Background

Ulcerative colitis (UC) is an inflammatory bowel disease (IBD) localized in the colon and rectum that is characterized by chronic and typically recurrent disease. Although the pathogenesis of UC has been confirmed to be related to genetic susceptibility, environmental factors and autoimmunity, it has not been fully elucidated [1, 2]. The typical clinical manifestations of UC are recurring abdominal pain, diarrhoea and bloody purulent stool. Severe UC may induce life-threatening complications, such as enterorrhagia and toxic megacolon [3]. The primary therapies used for mild and severe UC are limited to medication (corticosteroids, aminosalicylates and immunosuppressants) and surgical treatment [4, 5]. Under medical treatment, most patients with UC achieve temporary remission, while the long-term application of these drugs can trigger adverse effects, such as gastrointestinal reactions, hepatotoxicity nephrotoxicity and bone marrow suppression [6–8]. Surgical treatment is generally supposed to be the ultimate solution for UC, and ileal pouch anal anastomosis (IPAA) is a significant treatment for chronic and medically refractory mucosal UC. However, IPAA is accompanied by significant trauma and a variety of related complications, such as wound infection, anastomotic leakage or stricture, small bowel obstruction, pelvic sepsis, pouch-related fistula, pouchitis and pouch failure [9–11]. These side effects impose an economic burden on patients and seriously affect their quality of life [12]. Therefore, novel therapies for UC are urgently required to improve the quality of life of patients.

Mesenchymal stem cell (MSC) transplantation represents an innovative treatment for UC. MSCs have been shown to migrate to injuries at intestinal sites and facilitate damaged tissue repair by controlling the local development of inflammation [5, 13]. Dental pulp stem cells (DPSCs), a type of MSC, are characterized by self-renewal, multipotent differentiation potential and amplification in vitro. Under appropriate extracellular stimuli, DPSCs differentiate into various lineages, including osteoblasts, neurons, vascular cells and hepatocytes [14, 15]. Numerous studies have revealed that DPSCs could migrate to the lesion site, which accelerates tissue repair and regeneration [16, 17]. In addition, DPSCs present easy access, low-risk immune rejection and fewer ethical issues; hence, they can be used as ideal gene vehicles with wide application prospects [18, 19].

The intestinal mucosa is composed of proliferating epithelial cells. After intestinal mucosa injury occurs in UC, multiple growth factors and cytokines are induced at luminal and submucosal locations [20]. Research has shown that growth factors are strictly related to the processes of cell proliferation, migration, regeneration and ulcer healing [21]. Hepatocyte growth factor (HGF) secreted by MSCs plays a crucial role in the proliferation and migration of intestinal epithelial cells and reduces inflammatory cell infiltration [22]. Nevertheless, the physiological function of HGF is closely linked to the serum concentration, and repeated administration is required for a better therapeutic effect; however, such treatment is expensive and inconvenient and restrains the therapeutic effect on UC [22, 23]. Therefore, in this study, the rat HGF gene was transduced into DPSCs to compare the therapeutic effect of the HGF-DPSCs group with other treatment groups to determine whether HGF-overexpressing DPSCs provide the most suitable treatment for UC.

## Methods

### Experimental animals

Male Sprague-Dawley (SD) rats weighing approximately 100 g were purchased from the animal facility of the Second Affiliated Hospital of Harbin Medical University. All rats were maintained under 12:12-h light-dark cycles in standard animal cages and fed a standard pellet diet as well as drinking water ad libitum. All experiments and methods were performed strictly following the institutional guides for animal experiments, and they were approved by the Ethics Committee of the Second Affiliated Hospital of Harbin Medical University (No. SYDW2018-028).

### UC model establishment

The UC model was induced by intragastric administration [24, 25] using 5% dextran sulphate sodium (DSS, Shanghai Yuanye Bio-Technology Co., Ltd.) dissolved in distilled water (3.5 ml/100 g) for 14 consecutive days, while the controls were treated with distilled water (3.5 ml/100 g). To verify the establishment of the UC model, five DSS-treated experimental rats and five control rats were sacrificed to evaluate the changes in colon and rectum lengths, body weights and DAI scores. Then, the remaining DSS-treated rats were randomly divided into the following five groups (*n* = 5 rats/group): saline treatment (UC group, saline, 300 μl), HGF treatment (LV-HGF group, 300 μl), DPSCs treatment (DPSCs group, 1.0 × 10^6^ cells, 300 μl), green fluorescent protein (GFP)-modified DPSC treatment (GFP-DPSCs group, 1.0 × 10^6^ cells, 300 μl) and HGF-modified DPSCs treatment (HGF-DPSCs group, 1.0 × 10^6^ cells, 300 μl). All treatments were administered by tail vein injections into the rats.

### Haematoxylin-eosin staining

The colon tissues were collected and fixed in a 4% paraformaldehyde solution. After conventional dehydration and paraffin embedding, these tissues were cut into 5-μm-thick sections stained with haematoxylin-eosin (HE) according to the instructions. The slice images were observed by a BX51 microscope (Olympus, Tokyo, Japan).

### Evans blue staining

After using DSS for 14 consecutive days, five UC rats and control rats were randomly selected for Evans blue staining. Evans blue solution (2%, 4 ml/kg) was injected into rats through the tail vein. Then, the rats were sacrificed 2 h later, and intestinal staining was observed.

### Preparation of DPSCs for cell transplant therapy

DPSCs were extracted from the upper incisors of SD rats (male, 38–42 g, *n* = 2) and resuspended in phosphate-buffered saline (PBS) with 0.3% type I collagenase for 30 min at 37 °C. After centrifugation at 1000 rpm for 10 min, DPSCs were incubated with high-glucose DMEM containing 15% foetal bovine serum (FBS, ScienCell Research Laboratories, CA, USA), 100 IU/ml penicillin-G and 100 mg/ml streptomycin (JR Scientific, Woodland, CA) at 37 °C in a humidified atmosphere with 5% CO_2_. The third-generation DPSCs were transduced with lentiviral (LV)-HGF (Hanbio Biotechnology (Shanghai) Co., Ltd.) or LV-GFP at a multiplicity of infection (MOI) of 80 according to the manufacturer’s instructions. In short, the original culture medium of the DPSCs was removed via suction, and a 1/2 volume of lentivirus culture medium (0.5 ml) was added. Then, the cells were infected at 37 °C for 4 h and replenished to a normal volume by adding 0.5 ml culture medium. After infection for 24 h, the culture medium containing the virus was removed via suction, replaced with fresh complete medium and cultured at 37 °C. DPSCs with green fluorescence were observed under a fluorescence microscope after 48 h of infection. The LV-GFP-DPSCs and LV-HGF-DPSCs were screened by puromycin dihydrochloride (1 μg/ml, Thermo Fisher Scientific). The expression levels of HGF were assessed by western blotting.

### Flow cytometry

An immunophenotyping analysis of DPSCs was performed by flow cytometry. The cells were incubated with rat monoclonal antibodies against CD90 (0.2 mg/ml, 551401, BD Pharmingen), CD45 (0.2 mg/ml, 559135, BD Pharmingen), CD29 (0.5 mg/ml, 561796, BD Pharmingen) and CD11b (0.2 mg/ml, 562102, BD Pharmingen) at 4 °C for 30 min. An isotype control antibody was used as a negative control group. After incubation, DPSCs were washed by PBS. Signals were recorded by flow cytometry using a fluorescence-activated cell sorting (FACS) Canto II system (BD Biosciences, San Jose, CA, USA), and the data were analysed by FlowJo 10.0 (Tree Star, Inc., San Carlos, CA, USA).

### Osteogenic and adipogenic differentiation

The differentiation capacities of DPSCs were detected according to a previously described method [26]. Briefly, third-passage DPSCs were incubated with adipogenic or osteogenic differentiation medium for 2 weeks. Then, DPSCs were stained with oil red O (Sigma-Aldrich) and alizarin red S (Sigma-Aldrich) and observed under a microscope (Olympus, Tokyo, Japan).

### Tissue processing

After 4 weeks of treatment, the rats (*n* = 5) were anesthetized with xylazine (10 mg/kg) and ketamine (60 mg/kg). The colons were divided into three portions that were quickly frozen for nucleic acid and protein level detection, temporarily placed in 4% paraformaldehyde for histological analysis or provisionally placed in precooled PBS for oxidative stress markers determination. Additionally, the liver, spleen, kidney and lung tissues were also removed and temporarily placed in 4% paraformaldehyde for histological analysis.

### Immunofluorescence staining

The colon, liver, spleen, kidney and lung tissues of the rats (*n* = 5) were fixed in 4% paraformaldehyde overnight and then dehydrated in 30% sucrose solution. The tissues were embedded in optimal cutting temperature (OCT) compound and cut into 5-μm-thick frozen sections. After soaking with PBS, the sections were placed in 5% normal goat serum (abs933, Absin, Shanghai, China) and incubated with anti-Bmi1 (1:200, ab14389, Abcam), anti-Musashi1 (1:200, c-135,721, Santa Cruz Biotechnology), anti-Sox9 (1:100, ab3697, Abcam) and anti-PCNA antibodies (1:200, ab92552, Abcam) at 4 °C overnight. Next, the sections were washed in PBS and incubated at 37 °C for 1 h with anti-mouse IgG (1:500, 8890, Cell Signaling Technology) and anti-rabbit IgG (1:500, 8889, Cell Signaling Technology). Sections were then stained with DAPI and anti-fading medium before observation by a laser scanning confocal microscope (LSM 510 META; Zeiss, Germany), and the results were semi-quantitatively analysed with ImageJ (National Institutes of Health, Bethesda, USA).

### Immunohistochemical analysis

The colon tissues (*n* = 5) were embedded in paraffin and cut into 5-μm-thick slices after conventional dehydration. The sections were dewaxed in xylene and dehydrated in grade ethanol. These sections were placed in boiling ethylenediaminetetraacetic acid (EDTA) for antigen retrieval, and then 3% hydrogen peroxide was used to suppress endogenous peroxidase activity. After application of bovine serum albumin (BSA) for 30 min, the sections were incubated at 4 °C overnight with primary antibodies against Bmi1 (1:500, ab14389, Abcam), Musashi1 (1:200, sc-135721, Santa Cruz Biotechnology), Sox9 (1:200, ab3697, Abcam) and PCNA (1:200, ab92552, Abcam) followed by secondary antibodies (anti-rabbit (8114, Cell Signaling Technology) and anti-mouse (8125, Cell Signaling Technology)) for 1 h. Next, the sections were placed in diaminobenzidine (DAB) as the substrate and stained with haematoxylin. Then, the slides were subjected to conventional dehydration, clearing and sealing. The results were observed with a BX51 microscope (Olympus, Tokyo, Japan) and semiquantitatively analysed by ImageJ (National Institutes of Health, Bethesda, USA).

### Real-time quantitative PCR

Total RNA was extracted from frozen colon tissues using TRIzol reagent (Invitrogen, Carlsbad, CA, USA) according to the manufacturer’s instructions. The cDNAs were produced by reverse transcription using a Transcriptor First Strand cDNA Synthesis Kit (Roche Diagnostics GmbH, Mannheim, Germany). The template DNA was amplified by real-time quantitative PCR (RT-PCR) using the Fast Start Universal SYBR Green Master kit (Roche Diagnostics GmbH, Mannheim, Germany). In brief, PCRs were performed at 95 °C for 10 min to activate FastStart Taq DNA polymerase, followed by amplification of 40 cycles of 95 °C for 15 s and 60 °C for 1 min. The relative gene expression levels were normalized to β-actin using the 2^-ΔΔCT^ quantitation method [27]. The RT-PCR primers are shown below: TNF-α F: CGGAAAGCATGATCCGAGAT, R: AGACAGAAGAGCGTGGTGGC; IFN-γ F: GTGTCATCGAATCGCACCTGA, R: TTGTGCTGGATCTGTGGGTTG; TGF-β F: GAACCAAGGAGACGGAATACAGG, R: GAGGAGCAGGAAGGGTCGGT; IL-10 F: CCAGTCAGCCAGACCCACAT, R: GCATCACTTCTACCAGGTAAAAC; β-actin F: GGAGATTACTGCCCTGGCTCCTAGC, R: GGCCGGACTCATCGTACTCCTGCTT.

### Western blotting

Frozen colon tissues, DPSCs, GFP-DPSCs and HGF-DPSCs were homogenized in lysis buffer containing protease inhibitors. After the lysates were centrifuged at 12,000 rpm for 10 min at 4 °C, the supernatants were collected and the total protein concentration was measured by a BCA protein concentration determination kit (Beyotime, P00125) in accordance with the manufacturer’s instructions. Protein extracts were electrophoresed on 5% SDS-PAGE gels and further transferred to polyvinylidene fluoride (PVDF) membranes. After blocking with 5% skim milk for 1 h, the membranes were incubated with primary antibodies against TNF-ɑ (Santa Cruz Biotechnology, sc-52746, 1:500), TGF-β (Cell Signaling Technology, #3711, 1:500), IL-10 (Abcam, ab33471, 1:1000), IFN-γ (R&D Systems, MAB585, 1:1000), HGF (Abcam, ab83760, 1:500) and β-actin (Abcam, ab8226, 1:1000) at 4 °C overnight. Then, the membranes were washed in TBST three times and incubated with horseradish peroxidase (HRP)-conjugated anti-mouse (Abcam, ab6728, 1:5000) and anti-rabbit IgG (Abcam, ab6721, 1:5000) for 1 h at room temperature. The protein bands were visualized by enhanced chemiluminescence (ECL) solution, and the immunoblotting images were captured by an Omega-Lum G imaging system.

### Detection of oxidative stress

The colon tissues were washed thoroughly with precooled PBS (4 °C) and then homogenized and centrifuged at 5000 rpm for 10 min to obtain the supernatant, which was collected for myeloperoxidase (MPO, Cloud-Clone Corp, SEA601Ra), malondialdehyde (MDA, Cloud-Clone Corp, CEA597Ge) and superoxide dismutase (SOD, Cloud-Clone Corp, SES134Ra) assays. All procedures were performed according to the manufacturer’s instructions.

### Assessment of disease activity index

The disease activity index (DAI) scores were considered based on a complex evaluation of weight loss, stool consistency and bloody stool extent. Each parameter was assigned a score from 0 to 4, and the total score ranged from 0 (unaffected) to 12 (severe colitis) in accordance with previous studies [28, 29].

### Statistical analysis

All the data are presented as the means ± SD and were analysed by SPSS 24.0 (SPSS Inc., Chicago, IL, USA). Statistical analyses were performed by Student’s *t* test and one-way analysis of variance (one-way ANOVA) followed by Bonferroni’s multiple comparison test and two-way ANOVA. Statistical charts were prepared using GraphPad Prism 8.0 software (GraphPad Inc., La Jolla, CA, USA). Differences were identified as significant at *p* < 0.05.

## Results

### Verification of the rat model of UC

**Fig. 1:**
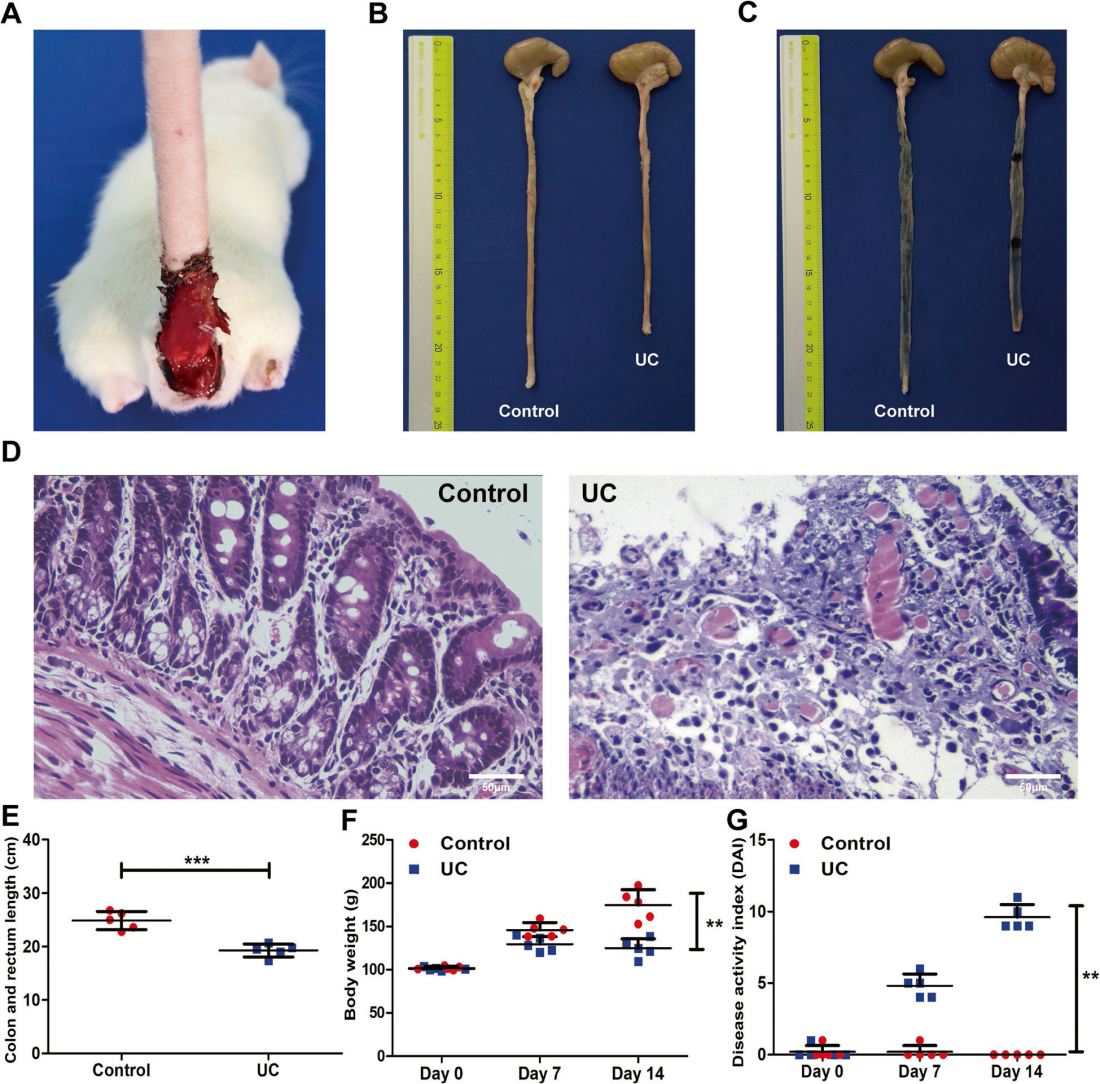
DSS-induced UC model in rats. **a** Rats had bloody stools after 5% DSS was administered for 14 days. **b**, **c** The colon and rectum lengths of the control and UC groups were compared, and changes after Evans blue staining were observed. **d** Comparison of HE staining between the control and UC groups (*n* = 5, scale bars = 50 μm). **e** Quantitative analysis of the colon and rectum length showed that the UC group was significantly shorter than the control group. Data are shown as the means ± SD (*n* = 5, ***p < 0.001). **f** Comparison of body weight changes between the control and UC group rats. Data are shown as the means ± SD (*n* = 5, **p < 0.01). **g** DAI scores were obtained by monitoring the body weight changes, stool consistency and bloody stool extent from the rats in the control and UC groups. Data are shown as the means ± SD (*n* = 5, **p < 0.01)

During the period of intragastric administration of DSS, weight alterations, faecal traits and haematochezia were constantly monitored. Over time, the rats showed bloody stool (Fig. 1a). Compared with the control group, the body weights of the UC group were dramatically lower at day 14, and the DAI scores showed the opposite trend (*p* < 0.01, Fig. 1f, g). The colon and rectum lengths of the UC group were significantly shorter than those of the control group (*p* < 0.001, Fig. 1b, e). Evans blue staining showed that the injury sites in the UC rats were darker than those in the control rats (Fig. 1c). Compared to the control group, a histopathology examination showed partially missing glands, mucosal epithelium necrosis and loss and a large number of infiltrating inflammatory cells in the UC group (Fig. 1d).

### Virus-transduced DPSCs exhibited mesenchymal stem cell antigenic markers

**Fig. 2:**
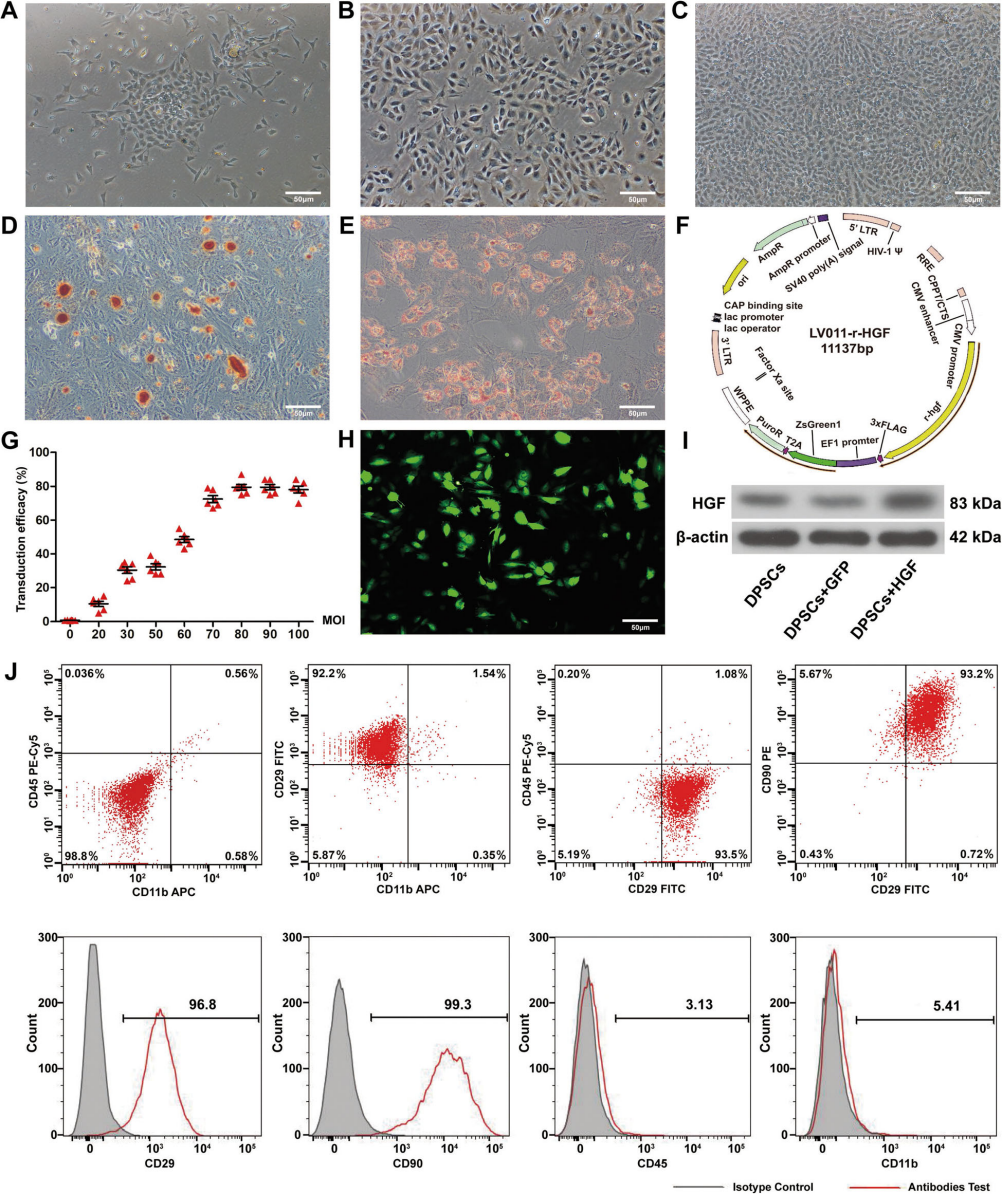
Virus-transduced DPSCs exhibited mesenchymal stem cell antigenic markers. **a**–**c** Morphology of the DPSCs. **d**–**e** Osteogenic and adipogenic differentiation of DPSCs. **f** Construction of the lentiviral vector to overexpress the rat HGF gene. **g** Relationship between the transduction efficiency and MOI of the virus. **h** HGF-DPSCs expressed green fluorescence under a microscope. Scale bar = 50 μm in all panels. **i** Western blotting analysis of DPSCs overexpressing HGF. **j** Specific antigenic markers of DPSCs were detected by flow cytometry (*n* = 3)

Under proper medium, DPSCs gradually attached to the culture flask wall and showed a fusiform shape (Fig. 2a–c). DPSCs transdifferentiated into adipocytes and osteocytes, which were identified by Oil Red O and Alizarin Red staining (Fig. 2d, e). The FACS results indicated that more than 95% of the DPSCs expressed CD90 and CD29, which are the antigenic phenotypes of MSCs, and less than 6% of the DPSCs expressed CD45 (haemopoietic stem cell marker) and CD11b (monocyte/macrophage surface marker) (Fig. 2j). Construction of the rat HGF gene-modified lentiviral vector is shown (Fig. 2f). The transduction efficiency was evaluated by different MOIs (Fig. 2g). Infected DPSCs were observed with bright green fluorescence under a laser scanning confocal microscope (Fig. 2h). The expression levels of HGF in HGF-DPSCs were the highest compared with DPSCs and GFP-DPSCs (Fig. 2i).

### Transplanted DPSCs homed to injured colons and transdifferentiated into intestinal stem cell-like cells

**Fig. 3:**
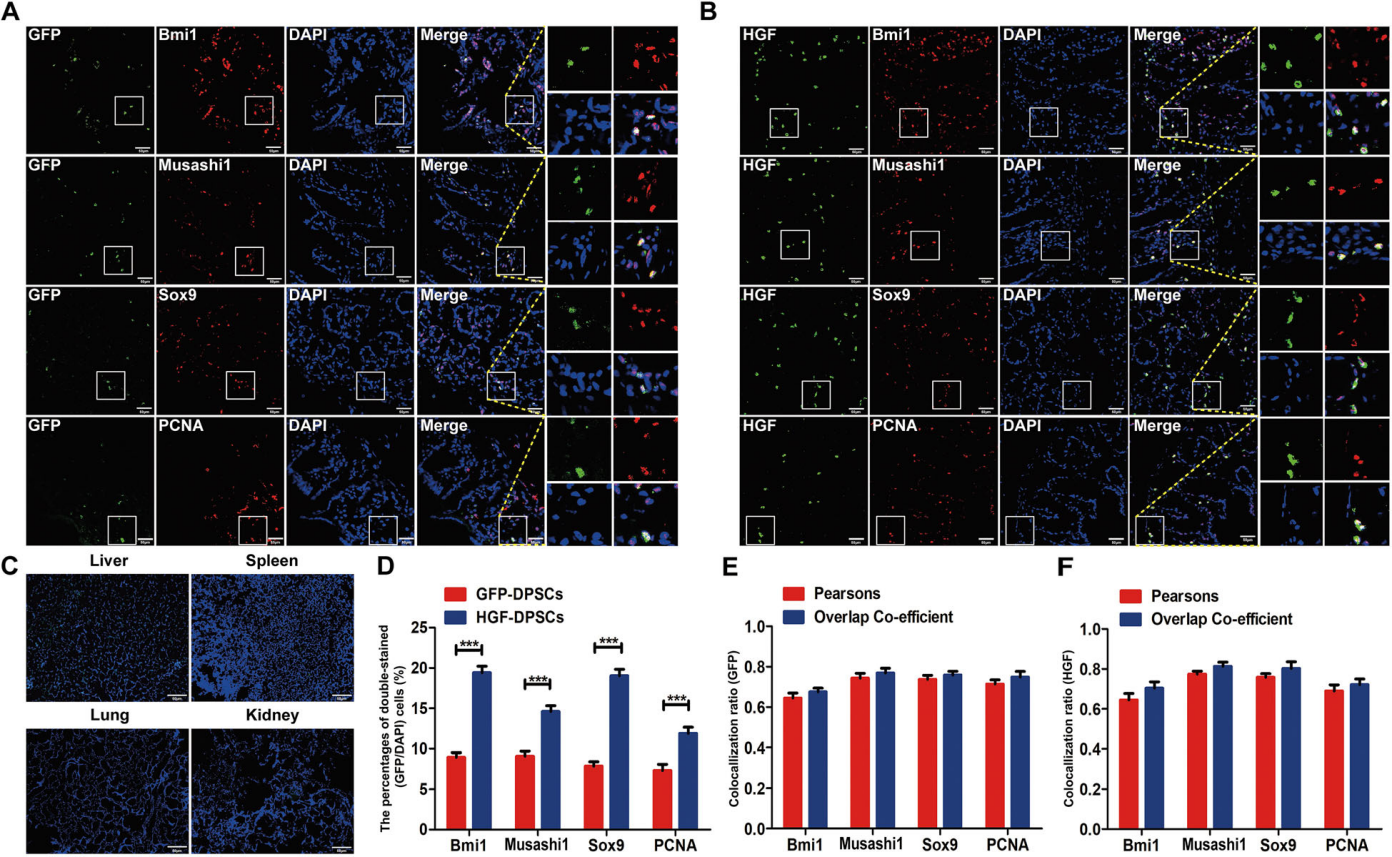
Transplanted DPSCs homed to injured colons and transdifferentiated into intestinal stem cell-like cells. **a**, **b** GFP-DPSCs and HGF-DPSCs expressing green fluorescence were colocalized with Bmi1, Musashi1, Sox9 and PCNA (*n* = 5). **c** Few positive cells were found in other organs of the rats (liver, spleen, kidney and lung tissues, *n* = 5). Scale bar = 50 μm in all panels. **d** Statistical comparison of the percentages of double-stained (GFP/DAPI) cells between the GFP-DPSCs and HGF-DPSCs groups. Data are shown as the means ± SD (*n* = 5; ***p < 0.001). **e**, **f** The comparison of Pearson’s correlation and the overlap coefficient of colon sections costained with Bmi1, Musashi1, Sox9 and PCNA between the GFP-DPSCs and HGF-DPSCs groups (*n* = 5; *p* > 0.05)

Four weeks after cell transplantation, immunofluorescence assays of Bmi1, Musashi1, Sox9 and PCNA were performed in the colon, liver, spleen, kidney and lung tissues. Bmi1, Musashi1 and Sox9 are intestinal stem cell (ISC) markers, including Bmi1, which was initially detected at the + 4 position from the bottom of the crypts [30, 31]. Musashi1 is strongly expressed at the base of the intestinal crypts [32]. Sox9 is widely expressed in the colonic crypts and intestinal epithelium and associated with ISC proliferation and self-renewal [33, 34]. PCNA is known as a proliferating cell nuclear antigen [35]. However, GFP-DPSCs and HGF-DPSCs were only visible in the colons (Fig. 3a–c). Moreover, GFP-DPSCs and HGF-DPSCs were colabelled with Bmi1, Musashi1, Sox9 and PCNA. A semiquantitative analysis showed that the percentages of double-stained (GFP/DAPI) cells in the HGF-DPSCs group were significantly higher than those in the GFP-DPSCs group (*p* < 0.01) (Fig. 3d). The GFP-DPSCs and HGF-DPSCs groups showed little difference in Pearson’s correlation coefficients and the overlap coefficients of the colon sections costained for Bmi1, Musashi1, Sox9 and PCNA (*p* > 0.05) (Fig. 3e, f).

### Transplanted DPSCs promoted ISC-like cell proliferation

**Fig. 4:**
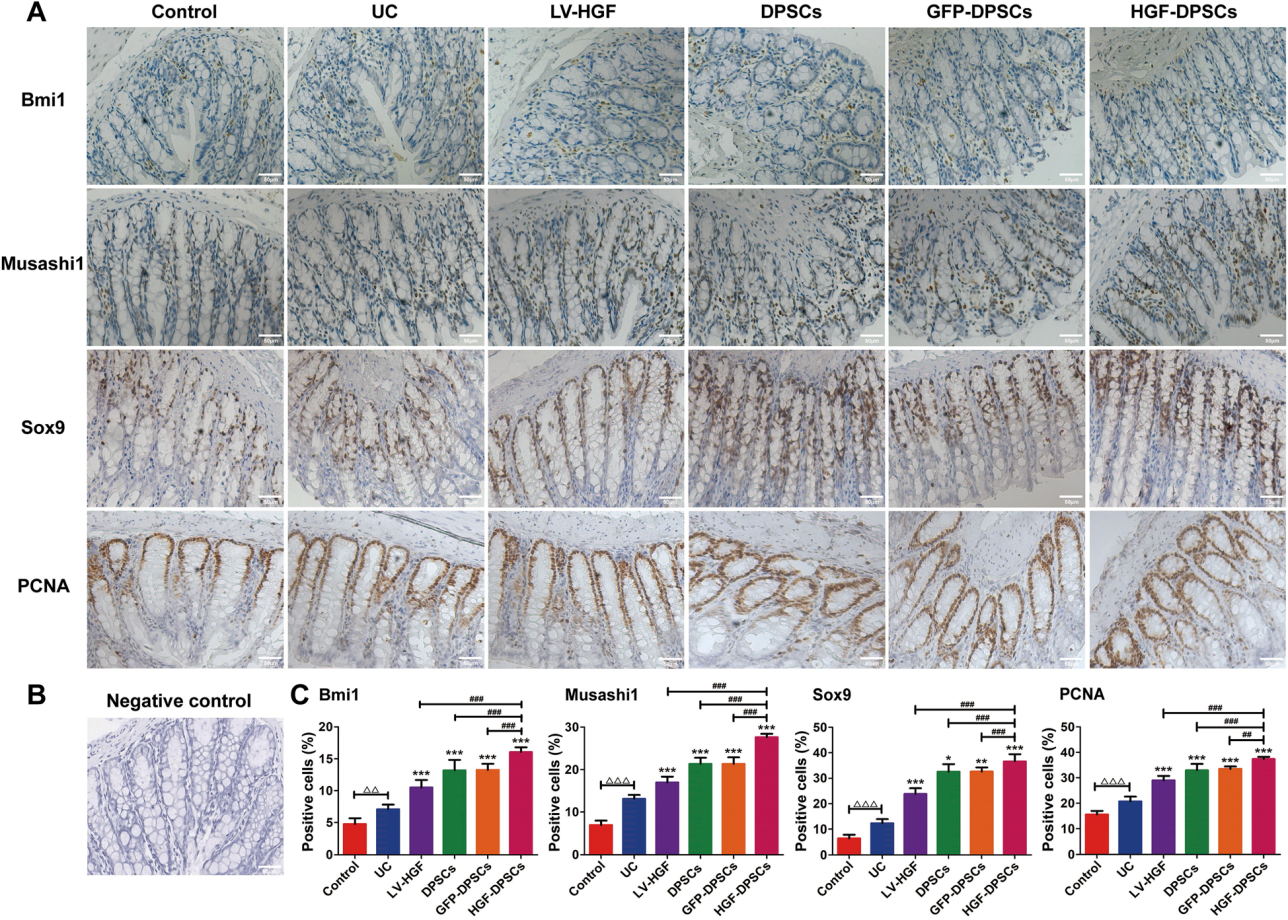
Transplanted DPSCs promoted ISC-like cell proliferation. **a** Increases in Bmi1-, Musashi1-, Sox9- and PCNA-positive cells were detected by immunohistochemical analysis. Scale bars = 50 μm. **b** The negative control result was staining without primary antibody. Scale bars = 50 μm. **c** Statistical comparison of the Bmi1-, Musashi1-, Sox9- and PCNA-positive cells in different groups. Data are shown as the means ± SD (*n* = 5; ^△△^*p* < 0.01, ^△△△^*p* < 0.001; *p < 0.05, **p < 0.01, ***p < 0.001 vs the UC group; ^##^*p* < 0.01, ^###^*p* < 0.001)

Immunohistochemistry analysis showed that the number of cells positive for Bmi1, Musashi1, Sox9 and PCNA was increased in the four treatment groups compared with the UC group (Fig. 4a). The negative control result is represented by staining without primary antibody (Fig. 4b). A quantitative analysis indicated that the number of positive cells in the control group was the lowest, whereas the number of these cells in the HGF-DPSCs group was the highest, which was significantly higher than that in the other treatment groups (*p* < 0.05). In addition, the positive cells in the DPSCs group were almost the same as those in the GFP-DPSCs group (Fig. 4c).

### Transplantation of DPSCs promoted injured colon tissue repair and suppressed intestinal inflammatory responses at the mRNA and protein levels

**Fig. 5:**
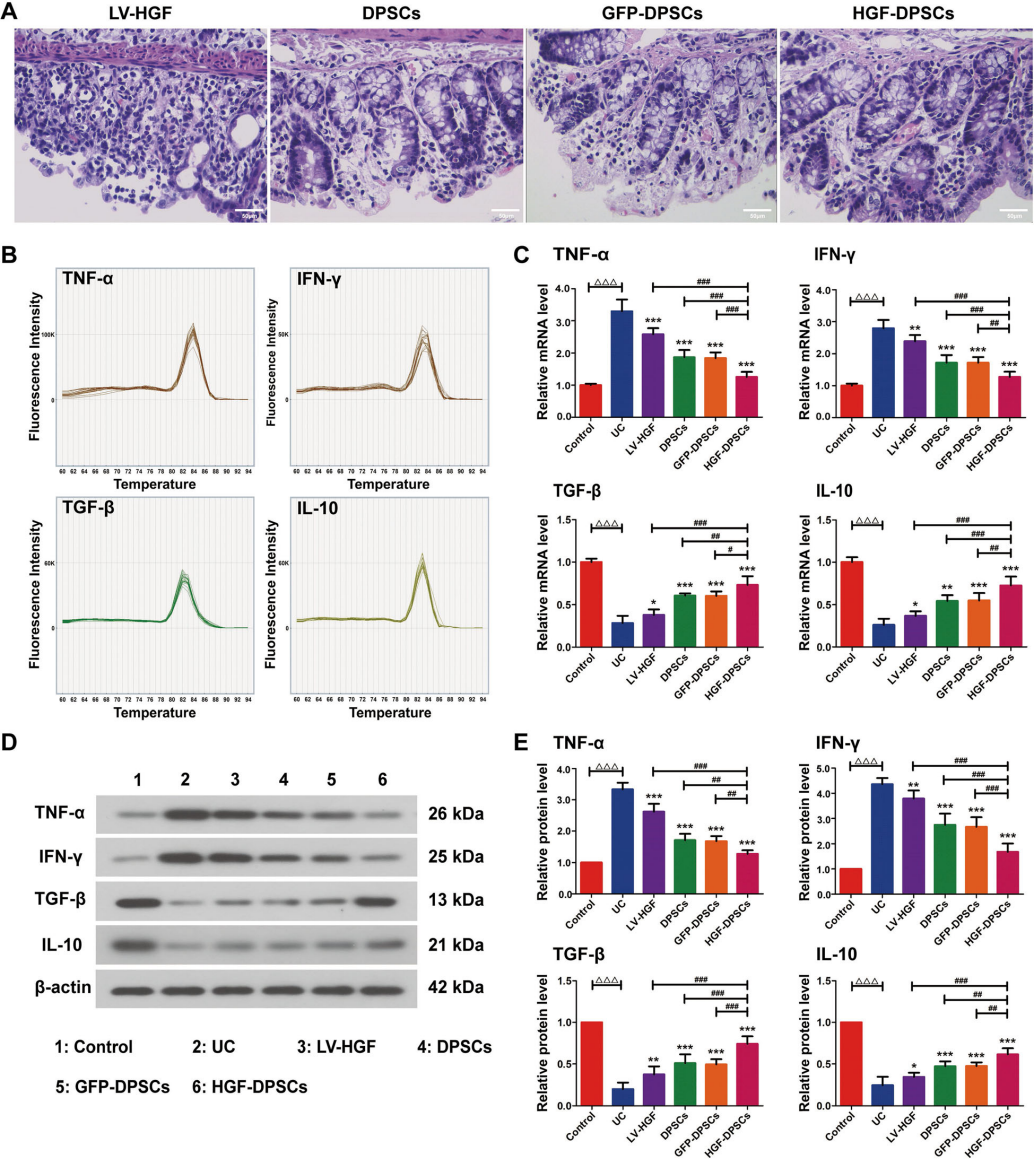
Transplantation of DPSCs promoted injured colon tissue repair and suppressed intestinal inflammatory responses at the mRNA and protein levels. **a** Transplanted DPSCs promoted the repair of damaged tissues. **b** The melting curves of TNF-α, IFN-γ, TGF-β and IL-10. **c** Statistical analysis of TNF-α, IFN-γ, TGF-β and IL-10 mRNA expression in rat colon tissues in different groups using RT-PCR. β-actin served as a reference. Data are shown as the means ± SD (*n* = 5; ^△△△^*p* < 0.001; *p < 0.05, **p < 0.01, ***p < 0.001 vs the UC group; ^#^*p* < 0.05, ^##^*p* < 0.01, ^###^*p* < 0.001). **d** Immunoblotting analysis of TNF-α, IFN-γ, TGF-β and IL-10 protein expression in rat colon tissues in different groups. **e** Quantitative analysis of TNF-α, IFN-γ, TGF-β and IL-10 expression. β-actin served as a reference. Data are shown as the means ± SD (*n* = 5; ^△△△^*p* < 0.001; *p < 0.05, **p < 0.01, ***p < 0.001 vs the UC group; ^##^*p* < 0.01, ^###^*p* < 0.001)

After 4 weeks of therapy, HE staining of colon tissues in the four treatment groups showed that transplanted DPSCs promoted the repair of damaged tissues (Fig. 5a). The mRNA and protein expression levels of the inflammatory cytokines in the colon in all groups were assayed. The melting curves of the four inflammatory cytokines all showed single peaks (Fig. 5b). The expression levels of proinflammatory cytokines (TNF-α and IFN-γ) were evidently decreased in the HGF-DPSCs group compared with those in the UC, LV-HGF, DPSCs and GFP-DPSCs groups (*p* < 0.05) (Fig. 5c–e). In contrast, the anti-inflammatory cytokines (TGF-β and IL-10) showed the opposite trend (*p* < 0.05) (Fig. 5c–e). In addition, limited differences in the expression of inflammatory cytokines were observed between the DPSCs and GFP-DPSCs groups (*p* > 0.05) (Fig. 5c–e).

### HGF-DPSCs suppressed oxidative stress responses

**Fig. 6:**
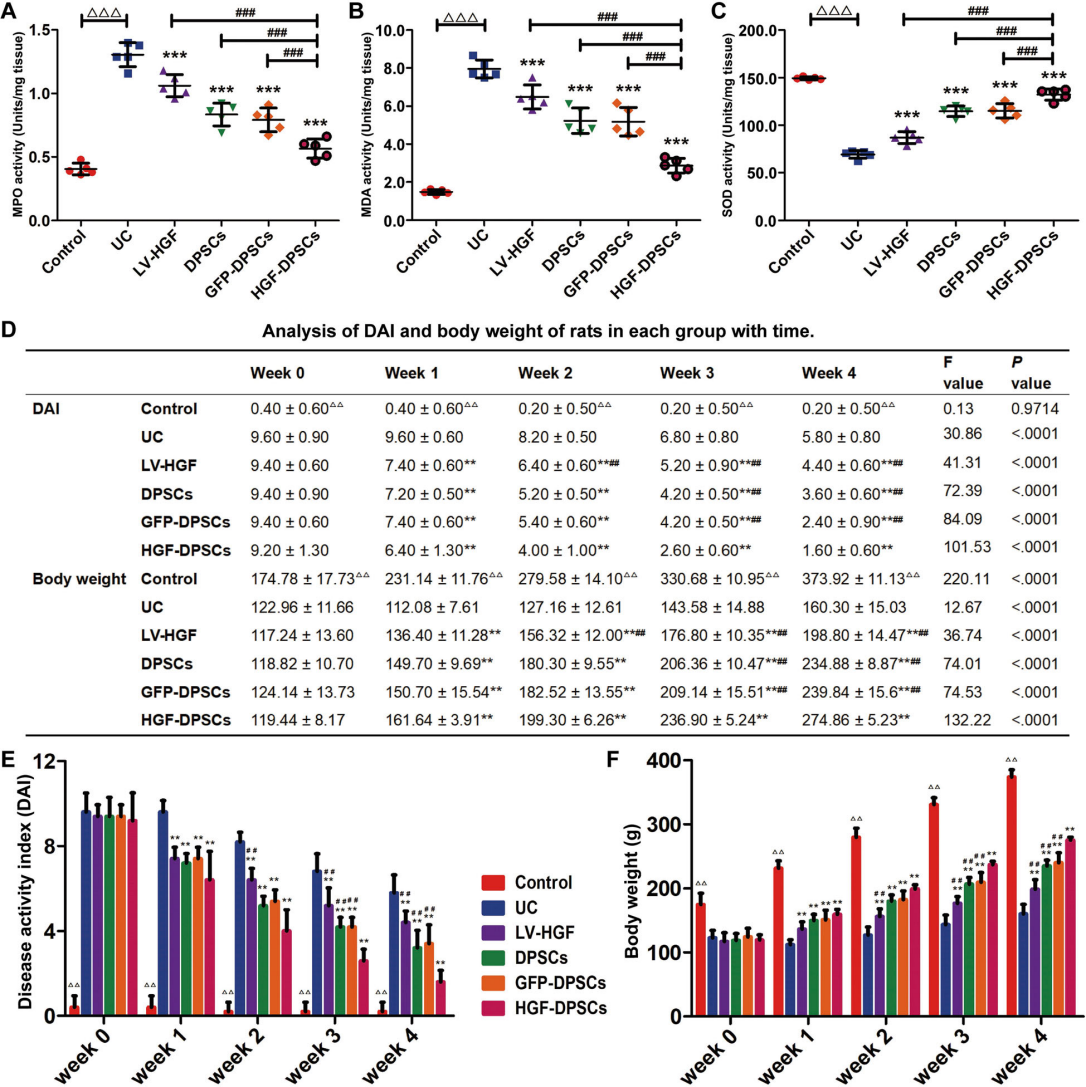
HGF-DPSCs suppressed oxidative stress responses and ameliorated DSS-induced disease activity. **a**–**c** Immunological detection of MPO, MDA and SOD in rat colon tissues from different groups. Data are shown as the means ± SD (*n* = 5; ^△△△^*p* < 0.001; ***p < 0.001 vs the UC group; ^###^*p* < 0.001). **d**–**f** Changes in the DAI and body weights of rats in different groups with prolonged time. Data are shown as the means ± SD (*n* = 5; ^△△^*p* < 0.01 vs the UC group; **p < 0.01 vs the UC group; ^##^*p* < 0.01 vs the HGF-DPSCs group)

To detect the oxidative stress responses in all groups, the MPO, MDA and SOD activities were assayed by an ELISA kit according to the manufacturer’s instructions. Statistical analysis indicated that the MPO and MDA activities were highest in the UC group and lowest in the control group (Fig. 6a, b). In the treatment groups, the HGF-DPSCs group expressed the lowest activity levels of MPO and MDA, and the expression values were significantly different from those of the HGF, DPSC and GFP-DPSCs groups (*p* < 0.001) (Fig. 6a, b). SOD activity followed the opposite trend (*p* < 0.001) (Fig. 6c). Additionally, the DPSCs and GFP-DPSCs groups showed little difference in activity expression.

### DSS-induced disease activity was alleviated by DPSCs

During the treatment period, the changes in DAI scores and body weights were recorded (Fig. 6d). Overall, the DAI scores showed a downward trend in the five treatment groups over 4 weeks, whereas the body weight increased. The reductions in DAI scores were most prominent in the HGF-DPSCs group. Moreover, at the 3rd and 4th weeks, the DAI scores of the HGF-DPSCs group were noticeably lower than those of the other treatment groups (*p* < 0.01) (Fig. 6e), while the body weights were higher than those of the other treatment groups (*p* < 0.01) (Fig. 6f).

## Discussion

UC is a chronic intestinal inflammatory disorder without an effective treatment strategy. Numerous studies have demonstrated that DPSCs transplantation might represent a potential therapy for UC [36, 37]. Additionally, HGF was reported to play a vital catalytic role in intestinal mucosal injury repair [38, 39]. Nevertheless, the therapeutic efficacy of DPSCs and HGF-DPSCs on UC is still unclear. Therefore, the rat-derived HGF gene was overexpressed in DPSCs by transduction of the lentiviral vector, and the therapeutic effects were explored in this study.

The DPSCs used in this study were extracted from rat incisors, and they expressed high amounts of MSC-specific markers (CD29 and CD90) and low amounts of MSC-nonspecific markers (CD45 and CD11b). Moreover, the multiple differentiation capacity was examined by osteogenesis and lipogenesis differentiation assessments, which were consistent with the defining criteria of MSCs [40]. After 4 weeks of tail vein injections in the rat UC model, GFP-DPSCs and HGF-DPSCs homed to the colon injury site, colocalized with ISC markers (Bmi1, Musashi1, Sox9) and proliferating cell nuclear antigen (PCNA) and significantly promoted the expression of these proteins. Under normal conditions, ISCs continue to proliferate, differentiate and self-renew and maintain the normal structure and function of the intestinal tract. Based on our results, we concluded that GFP-DPSCs and HGF-DPSCs relieve colon injury by transdifferentiation into ISC-like cells and accelerating ISC-like cell proliferation. In addition, our results also found that the number of HGF-DPSCs reaching the colon injury site was significantly higher than that of GFP-DPSCs, which may be because HGF-DPSCs promoted the ability of DPSCs to home to the injury site.

To further explore the mechanism of HGF-DPSCs in treating UC, oxidative stress indexes (MPO, MDA and SOD) were assessed. Oxidative stress is an essential factor in promoting the occurrence and progression of UC. In the process of injury and repair of UC, the inflammatory response and oxidative stress complement each other. The massive release of inflammatory cytokines causes oxidative stress damage, which further exacerbates the inflammatory response in active UC [41, 42]. The oxidative stress response directly or indirectly damages intestinal epithelial cells and destroys the integrity of the mucosal barrier, which is also an essential mechanism in UC [43]. In our study, the activity of MPO and MDA was decreased, while the activity of SOD was increased in the treatment groups, especially in the HGF-DPSCs group. MPO activity can reflect the degree of neutrophil infiltration, indicating that there may be significant tissue damage in UC [44–46]. MDA is closely related to the level of oxygen free radicals and is a marker of lipid peroxidation damage; it is also a biochemical link between oxidative stress and inflammation [41, 47]. SOD is an antioxidant that degrades reactive oxygen species and prevents some cells from undergoing peroxidation [48, 49]. Similar results were observed in the examinations of inflammatory cytokines. Pro-inflammatory cytokine (TNF-α, INF-γ) expression levels were remarkably reduced in the treatment groups, while anti-inflammatory cytokine (TGF-β, IL-10) expression showed the opposite trend. In addition, the HGF-DPSCs group had the highest expression level of anti-inflammatory cytokines and the lowest expression of pro-inflammatory cytokines. Therefore, we speculated that HGF-DPSCs ameliorated UC by inhibiting the oxidative stress response and inflammatory response. Moreover, cells lacking *Bmi1* were reported to cause mitochondrial dysfunction and the accumulation of reactive oxygen species [31, 50]. Interestingly, the expression of Bmi1 was significantly increased in the HGF-DPSCs group, which may be a potential mechanism underlying the effect of HGF-DPSCs on UC. Although this study has confirmed that the therapeutic effect of HGF-DPSCs is better than that of DPSCs, the transdifferentiation efficiency of HGF and DPSCs has not been measured specifically and thus needs to be further explored by subsequent experiments.

## Conclusions

In summary, our study revealed that HGF-DPSCs have a good therapeutic effect on a rat UC model. HGF-DPSCs dramatically relieved disease activity by transdifferentiating into ISC-like cells, promoting ISC-like cell proliferation, suppressing the inflammatory response and reducing oxidative stress damage.

## Supplementary Information

**Additional file 1: Supplementary Fig. 1**. Gating strategy. **(A)** Cell distribution figure. Close to the axis are the cell fragments, circled cells that need to be analysed. **(B)** Double staining of CD29 and CD90. The horizontal and vertical coordinates are the fluorescence intensities of CD29 and CD90, respectively. **(C)** Double staining of CD45 and CD11b. The horizontal and vertical coordinates are the fluorescence intensities of CD45 and CD11b, respectively. **(D-G)** The histograms of CD29, CD90, CD45 and CD11b were obtained by circling the positive cells according to the isotype control.

## Data Availability

Please contact the corresponding author for data requests.

## Abbreviations

UC: Ulcerative colitis
IBD: Inflammatory bowel disease
MSCs: Mesenchymal stem cells
DPSCs: Dental pulp stem cells
HGF: Hepatocyte growth factor
SD: Sprague-Dawley
DSS: Dextran sulphate sodium
HE: Haematoxylin-eosin
PBS: Phosphate-buffered saline
LV: Lentiviral
FACS: Fluorescence-activated cell sorting
OCT: Optimal cutting temperature
EDTA: Ethylene diamine tetraacetic acid
DAB: Diaminobenzidine
PVDF: Polyvinylidene fluoride
ECL: Enhanced chemiluminescence
MPO: Myeloperoxidase
MDA: Malondialdehyde
SOD: Superoxide dismutase
DAI: Disease activity index
ANOVA: Analysis of variance
MOI: Multiple of infection
ISCs: Intestinal stem cells

## References

[1] Baumgart DC, Carding SR (2007). Inflammatory bowel disease: cause and immunobiology. Lancet.

[2] Chen X, Zhao X, Wang H, Yang Z, Li J, Suo H. Prevent effects of Lactobacillus fermentum HY01 on dextran sulfate sodium-induced colitis in mice. Nutrients. 2017;9. 10.3390/nu9060545. 10.3390/nu9060545 PMC5490524 28587089

[3] Zong S, Pu Y, Li S, Xu B, Zhang Y, Zhang T, Wang B. Beneficial anti-inflammatory effect of paeonol self-microemulsion-loaded colon-specific capsules on experimental ulcerative colitis rats. Artif Cells Nanomed Biotechnol 2018;46:324–335. doi:10.1080/21691401.2017.1423497. 10.1080/21691401.2017.1423497 29316822

[4] Kornbluth A, Sachar DB (2010). Practice Parameters Committee of the American College of Gastroenterology. Ulcerative colitis practice guidelines in adults: American College Of Gastroenterology, Practice Parameters Committee. Am J Gastroenterol.

[5] Robinson AM, Rahman AA, Miller S, Stavely R, Sakkal S, Nurgali K (2017). The neuroprotective effects of human bone marrow mesenchymal stem cells are dose-dependent in TNBS colitis. Stem Cell Res Ther.

[6] Allison J, Herrinton LJ, Liu L, Yu J, Lowder J (2008). Natural history of severe ulcerative colitis in a community-based health plan. Clin Gastroenterol Hepatol.

[7] Forte D, Ciciarello M, Valerii MC, De Fazio L, Cavazza E, Giordano R, Parazzi V, Lazzari L, Laureti S, Rizzello F, Cavo M, Curti A, Lemoli RM, Spisni E, Catani L (2015). Human cord blood-derived platelet lysate enhances the therapeutic activity of adipose-derived mesenchymal stromal cells isolated from Crohn’s disease patients in a mouse model of colitis. Stem Cell Res Ther.

[8] Keane TJ, Dziki J, Sobieski E, Smoulder A, Castleton A, Turner N, White LJ, Badylak SF (2017). Restoring mucosal barrier function and modifying macrophage phenotype with an extracellular matrix hydrogel: potential therapy for ulcerative colitis. J Crohns Colitis.

[9] Fazio VW, Kiran RP, Remzi FH, Coffey JC, Heneghan HM, Kirat HT, Manilich E, Shen B, Martin ST (2013). Ileal pouch anal anastomosis: analysis of outcome and quality of life in 3707 patients. Ann Surg.

[10] Henriksen M, Jahnsen J, Lygren I, Sauar J, Kjellevold Ø, Schulz T, Vatn MH, Moum B, IBSEN Study Group (2006). Ulcerative colitis and clinical course: results of a 5-year population-based follow-up study (the IBSEN study). Inflamm Bowel Dis.

[11] Kiely JM, Fazio VW, Remzi FH, Shen B, Kiran RP (2012). Pelvic sepsis after IPAA adversely affects function of the pouch and quality of life. Dis Colon Rectum.

[12] Sun T, Gao GZ, Li RF, Li X, Li DW, Wu SS, Yeo AE, Jin B (2015). Bone marrow-derived mesenchymal stem cell transplantation ameliorates oxidative stress and restores intestinal mucosal permeability in chemically induced colitis in mice. Am J Transl Res.

[13] Sala E, Genua M, Petti L, Anselmo A, Arena V, Cibella J (2015). Mesenchymal stem cells reduce colitis in mice via release of TSG6, independently of their localization to the intestine. Gastroenterology.

[14] Yamada Y, Fujimoto A, Ito A, Yoshimi R, Ueda M (2006). Cluster analysis and gene expression profiles: a cDNA microarray system-based comparison between human dental pulp stem cells (hDPSCs) and human mesenchymal stem cells (hMSCs) for tissue engineering cell therapy. Biomaterials.

[15] Zhang C, Zhang Y, Feng Z, Zhang F, Liu Z, Sun X, Ruan M, Liu M, Jin S (2018). Therapeutic effect of dental pulp stem cell transplantation on a rat model of radioactivity-induced esophageal injury. Cell Death Dis.

[16] Jin Q, Yuan K, Lin W, Niu C, Ma R, Huang Z (2019). Comparative characterization of mesenchymal stem cells from human dental pulp and adipose tissue for bone regeneration potential. Artif Cells Nanomed Biotechnol..

[17] Martínez-Sarrà E, Montori S, Gil-Recio C, Núñez-Toldrà R, Costamagna D, Rotini A, Atari M, Luttun A, Sampaolesi M (2017). Human dental pulp pluripotent-like stem cells promote wound healing and muscle regeneration. Stem Cell Res Ther.

[18] Alsaeedi HA, Koh AE, Lam C, Rashid M, Harun M, Saleh M (2019). Dental pulp stem cells therapy overcome photoreceptor cell death and protects the retina in a rat model of sodium iodate-induced retinal degeneration. J Photochem Photobiol B.

[19] Yamada Y, Nakamura-Yamada S, Kusano K, Baba S. Clinical potential and current progress of dental pulp stem cells for various systemic diseases in regenerative medicine: a concise review. Int J Mol Sci. 2019;20. 10.3390/ijms20051132. 10.3390/ijms20051132 PMC6429131 30845639

[20] Numata M, Ido A, Moriuchi A, Kim I, Tahara Y, Yamamoto S, Hasuike S, Nagata K, Miyata Y, Uto H, Tsubouchi H (2005). Hepatocyte growth factor facilitates the repair of large colonic ulcers in 2,4,6-trinitrobenzene sulfonic acid-induced colitis in rats. Inflamm Bowel Dis.

[21] Tahara Y, Ido A, Yamamoto S, Miyata Y, Uto H, Hori T, Hayashi K, Tsubouchi H (2003). Hepatocyte growth factor facilitates colonic mucosal repair in experimental ulcerative colitis in rats. J Pharmacol Exp Ther.

[22] Hanawa T, Suzuki K, Kawauchi Y, Takamura M, Yoneyama H, Han GD, Kawachi H, Shimizu F, Asakura H, Miyazaki J, Maruyama H, Aoyagi Y (2006). Attenuation of mouse acute colitis by naked hepatocyte growth factor gene transfer into the liver. J Gene Med.

[23] Li J, Zheng CQ, Li Y, Yang C, Lin H, Duan HG (2015). Hepatocyte growth factor gene-modified mesenchymal stem cells augment sinonasal wound healing. Stem Cells Dev.

[24] Tran CD, Ball JM, Sundar S, Coyle P, Howarth GS (2007). The role of zinc and metallothionein in the dextran sulfate sodium-induced colitis mouse model. Dig Dis Sci.

[25] Wang R, Wu G, Du L, Shao J, Liu F, Yang Z, Liu D, Wei Y (2016). Semi-bionic extraction of compound turmeric protects against dextran sulfate sodium-induced acute enteritis in rats. J Ethnopharmacol.

[26] Di Scipio F, Sprio AE, Carere ME, Yang Z, Berta GN (2017). A simple protocol to isolate, characterize, and expand dental pulp stem cells. Methods Mol Biol.

[27] Livak KJ, Schmittgen TD (2001). Analysis of relative gene expression data using real-time quantitative PCR and the 2(-Delta Delta C(T)) method. Methods.

[28] Arab HH, Al-Shorbagy MY, Abdallah DM, Nassar NN (2014). Telmisartan attenuates colon inflammation, oxidative perturbations and apoptosis in a rat model of experimental inflammatory bowel disease. PLoS One.

[29] Zhang ZL, Fan HY, Yang MY, Zhang ZK, Liu K (2014). Therapeutic effect of a hydroxynaphthoquinone fraction on dextran sulfate sodium-induced ulcerative colitis. World J Gastroenterol.

[30] Roth S, Franken P, Sacchetti A, Kremer A, Anderson K, Sansom O, Fodde R (2012). Paneth cells in intestinal homeostasis and tissue injury. PLoS One.

[31] López-Arribillaga E, Rodilla V, Pellegrinet L, Guiu J, Iglesias M, Roman AC, Gutarra S, González S, Muñoz-Cánoves P, Fernández-Salguero P, Radtke F, Bigas A, Espinosa L (2015). Bmi1 regulates murine intestinal stem cell proliferation and self-renewal downstream of Notch. Development.

[32] Luo J, Cao J, Jiang X, Cui H (2010). Effect of low molecular weight heparin rectal suppository on experimental ulcerative colitis in mice. Biomed Pharmacother.

[33] Davidson LA, Goldsby JS, Callaway ES, Shah MS, Barker N, Chapkin RS (2012). Alteration of colonic stem cell gene signatures during the regenerative response to injury. Biochim Biophys Acta.

[34] Flandez M, Guilmeau S, Blache P, Augenlicht LH (2008). KLF4 regulation in intestinal epithelial cell maturation. Exp Cell Res.

[35] Choi YJ, Choi YJ, Kim N, Nam RH, Lee S, Lee HS, Lee HN, Surh YJ, Lee DH (2017). Açaí berries inhibit colon tumorigenesis in azoxymethane/dextran sulfate sodium-treated mice. Gut Liver.

[36] Földes A, Kádár K, Kerémi B, Zsembery Á, Gyires K, Zádori ZS, Varga G (2016). Mesenchymal stem cells of dental origin-their potential for antiinflammatory and regenerative actions in brain and gut damage. Curr Neuropharmacol.

[37] Lei M, Li K, Li B, Gao LN, Chen FM, Jin Y (2014). Mesenchymal stem cell characteristics of dental pulp and periodontal ligament stem cells after in vivo transplantation. Biomaterials.

[38] Yuge K, Takahashi T, Khai NC, Goto K, Fujiwara T, Fujiwara H, Kosai K (2014). Intramuscular injection of adenoviral hepatocyte growth factor at a distal site ameliorates dextran sodium sulfate-induced colitis in mice. Int J Mol Med.

[39] Ando Y, Inaba M, Sakaguchi Y, Tsuda M, Quan GK, Omae M, Okazaki K, Ikehara S (2008). Subcutaneous adipose tissue-derived stem cells facilitate colonic mucosal recovery from 2,4,6-trinitrobenzene sulfonic acid (TNBS)-induced colitis in rats. Inflamm Bowel Dis.

[40] Dominici M, Le Blanc K, Mueller I, Slaper-Cortenbach I, Marini F, Krause D, Deans R, Keating A, Dj P, Horwitz E (2006). Minimal criteria for defining multipotent mesenchymal stromal cells. The International Society for Cellular Therapy position statement. Cytotherapy.

[41] Wang D, Zhang Y, Yang S, Zhao D, Wang M (2019). A polysaccharide from cultured mycelium of Hericium erinaceus relieves ulcerative colitis by counteracting oxidative stress and improving mitochondrial function. Int J Biol Macromol.

[42] Benhar M. Oxidants, antioxidants and thiol redox switches in the control of regulated cell death pathways. Antioxidants (Basel). 2020;9. 10.3390/antiox9040309. 10.3390/antiox9040309 PMC7222211 32290499

[43] El Sayed NS, Sayed AS. Protective effect of methylene blue on TNBS-induced colitis in rats mediated through the modulation of inflammatory and apoptotic signalling pathways. Arch Toxicol. 2019. 10.1007/s00204-019-02548-w. 10.1007/s00204-019-02548-w 31428839

[44] Lv Y, Yang X, Huo Y, Tian H, Li S, Yin Y, Hao Z (2012). Adenovirus-mediated hepatocarcinoma-intestine-pancreas/pancreatitis-associated protein suppresses dextran sulfate sodium-induced acute ulcerative colitis in rats. Inflamm Bowel Dis.

[45] Sánchez-Fidalgo S, Villegas I, Aparicio-Soto M, Cárdeno A, Rosillo MÁ, González-Benjumea A, Marset A, López Ó, Maya I, Fernández-Bolaños JG, de la Lastra CA (2015). Effects of dietary virgin olive oil polyphenols: hydroxytyrosyl acetate and 3, 4-dihydroxyphenylglycol on DSS-induced acute colitis in mice. J Nutr Biochem.

[46] Yan YX, Shao MJ, Qi Q, Xu YS, Yang XQ, Zhu FH, He SJ, He PL, Feng CL, Wu YW, Li H, Tang W, Zuo JP (2018). Artemisinin analogue SM934 ameliorates DSS-induced mouse ulcerative colitis via suppressing neutrophils and macrophages. Acta Pharmacol Sin.

[47] Amirshahrokhi K, Bohlooli S, Chinifroush MM (2011). The effect of methylsulfonylmethane on the experimental colitis in the rat. Toxicol Appl Pharmacol.

[48] Kang JE, Kim HD, Park SY, Pan JG, Kim JH, Yum DY (2018). Dietary supplementation with a Bacillus superoxide dismutase protects against γ-radiation-induced oxidative stress and ameliorates dextran sulphate sodium-induced ulcerative colitis in mice. J Crohns Colitis.

[49] Moura FA, de Andrade KQ, de Araújo OR, Nunes-Souza V, Santos JC, Rabelo LA, Goulart MO (2016). Colonic and hepatic modulation by lipoic acid and/or N-acetylcysteine supplementation in mild ulcerative colitis induced by dextran sodium sulfate in rats. Oxidative Med Cell Longev.

[50] Nakamura S, Oshima M, Yuan J, Saraya A, Miyagi S, Konuma T, Yamazaki S, Osawa M, Nakauchi H, Koseki H, Iwama A (2012). Bmi1 confers resistance to oxidative stress on hematopoietic stem cells. PLoS One.

