# Supplementary figures and images for: Dental pulp stem cells overexpressing hepatocyte growth factor facilitate the repair of DSS-induced ulcerative colitis
Source: Stem Cell Res Ther. 2021 Jan 7;12:30. doi: 10.1186/s13287-020-02098-4 (PMC7792189; doi:10.1186/s13287-020-02098-4)

**A**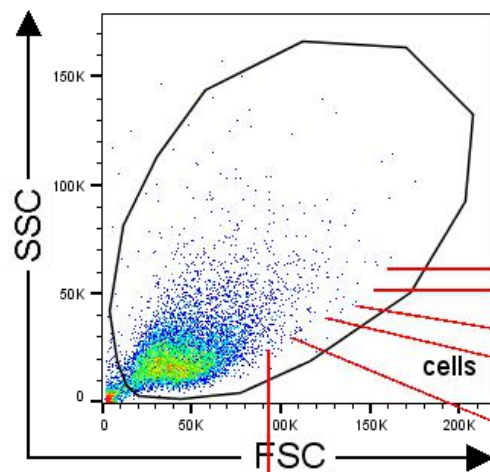**B**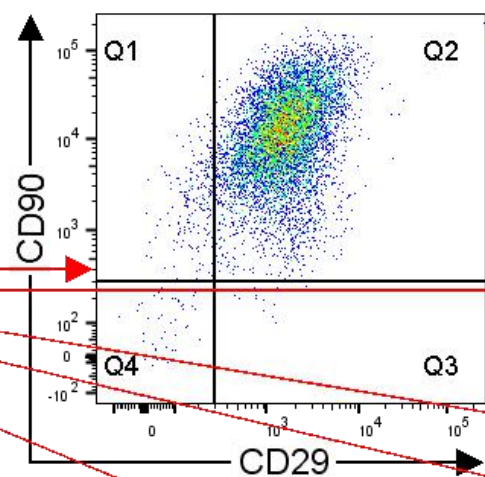**C**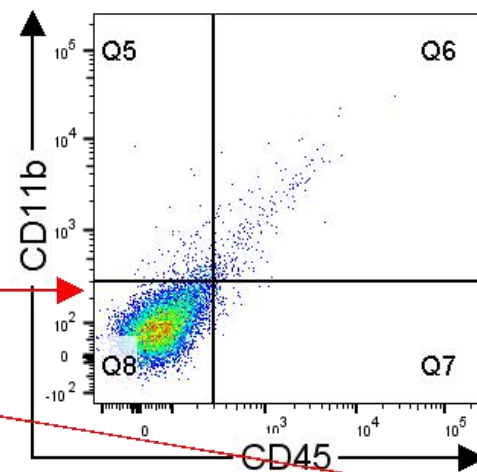**D**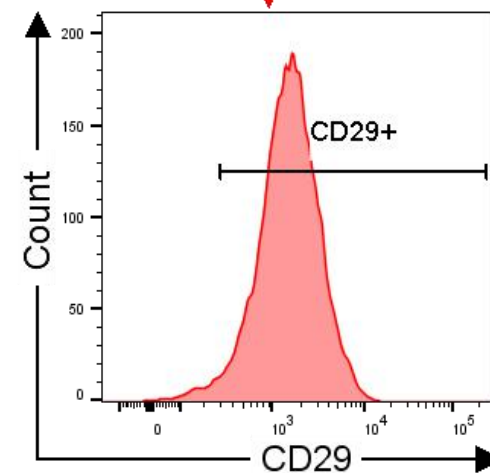**E**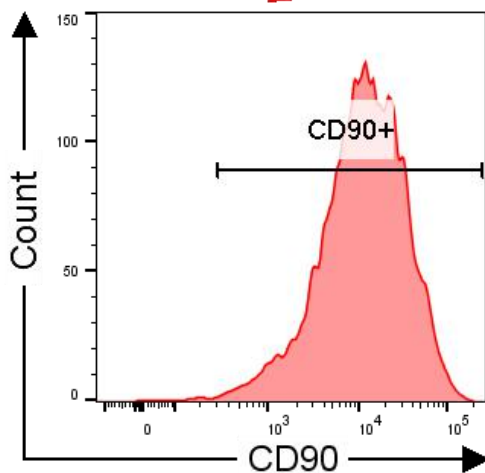**F**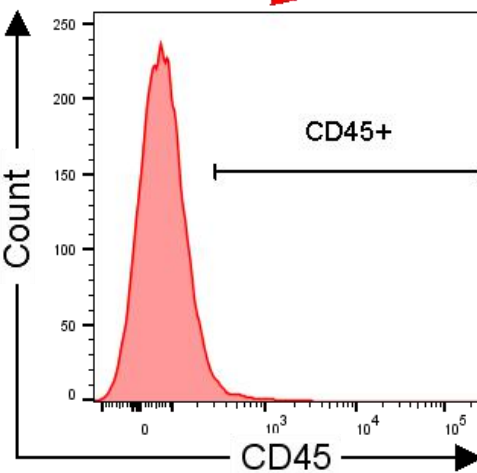**G**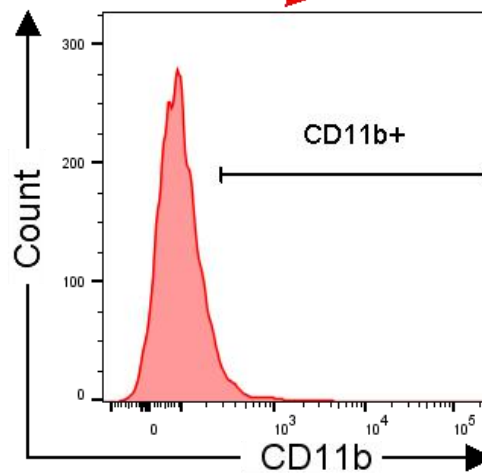

Supplement: Supplementary file 1 — Additional file 1: Supplementary Fig. 1. Gating strategy. (A) Cell distribution figure. Close to the axis are the cell fragments, circled cells that need to be analysed. (B) Double staining of CD29 and CD90. The horizontal and vertical coordinates are the fluorescence intensities of CD29 and CD90, respectively. (C) Double staining of CD45 and CD11b. The horizontal and vertical coordinates are the fluorescence intensities of CD45 and CD11b, respectively. (D-G) The histograms of CD29, CD90, CD45 and CD11b were obtained by circling the positive cells according to the isotype control. [file 13287_2020_2098_MOESM1_ESM.pdf]
